# Prospects of Apicultural Entrepreneurship in Coastal Districts of Eastern India: A Melissopalynological Evaluation

**DOI:** 10.1371/journal.pone.0094572

**Published:** 2014-04-16

**Authors:** Debasis Upadhyay, Swapan Bhattacharya, David K. Ferguson, Subir Bera

**Affiliations:** 1 Department of Botany, Budge Budge College, Kolkata, West Bengal, India; 2 Department of Microbiology, Moulana Azad College, Kolkata, West Bengal, India; 3 Department of Paleontology, University of Vienna, Vienna, Austria; 4 Centre of Advanced Studies, Department of Botany, University of Calcutta, West Bengal, India; Universidade de São Paulo, Faculdade de Filosofia Ciências e Letras de Ribeirão Preto, Brazil

## Abstract

A melissopalynological analysis of fifty-one natural honey samples (twenty four spring, fifteen summer and twelve winter) collected during 2010–2011 from two east-coastal districts (20^0^20^/^ to 22^0^11^/^ N, 82^0^39^/^ to 87^0^01^/^ E) of Orissa, India was performed. Out of 37 unifloral samples found 25 were contributed by *Apis cerana indica*, seven by *A. dorsata* and the remaining five by *A. florea*. Out of 14 multifloral samples five were contributed by *A. cerana indica*, five by *A. dorsata* and the remaining four by *A. florea*. Principal component analysis confirmed the palynological classification of the unifloral honey samples. Eighty-two bee-plant taxa belonging to forty four families were recovered. The predominant nectariferous taxa of the spring season were *Acanthus ilicifolius, Avicennia marina, Bruguiera gymnorrhiza, Cocos nucifera, Eucalyptus globulus, Phoenix paludosa, Pongamia pinnata, Prosopis juliflora*, *Sonneratia apetala* and *Syzygium cumini*. In the summer the predominant nectariferous taxa were *Borassus flabellifer, C. nucifera, E. globulus, Syzygium cumini, Terminalia arjuna, Aegiceras corniculatum, P. paludosa* and *Sonneratia apetala* while those of the winter were *Brassica nigra, Coriandrum sativum, Zizyphus jujuba, Alstonia scholaris, E. globulus and Bruguiera gymnorrhiza*. Very low (<0.09) HDE/P for 98% of the samples and absence of toxic palynotaxa assure that these honeys are suitable for human consumption. Quite extended honey flow period with spring and summer as best forage seasons for the honeybees and occurrence of 82% of these honeys with APC Group II, III and IV justify the sustainability of the present study area for establishing moderate to large-scale apicultural entrepreneurship. This should improve the socio-economic status of the people of this region.

## Introduction

Honey is produced by mutual interactions between bees and nectariferous plants. Microscopic analysis of pollen grains of natural honeys known as melissopalynology, allows identification of the different nectar sources over the season. Bees use these sources for the production of honey in a region, classifying the honey botanically and geographically according to its origin [1], [2], [3]. Melissopalynology is therefore extremely useful for hive management and allows the identification of likely periods of production of unifloral honey (honey from only one floral source), which has high commercial value [4], [5]. Usually the beekeepers do not have information of all the important nectar plants contributing to honey production and thus pollen analysis proves to be a useful guide to beekeeping in a region [6].

The per capita income of people in the coastal state of Orissa, is among those of the seven poorest states of India during 2010–2011 which amounts to $ 600 as against the Indian national figure of $ 1000 [7]. Moreover during 2009–2010 the rural poverty line of this state was as low as approximately $ 0.30 per day, against the urban poverty line $ 0.40 per day [8]. Tribal populations and forest dwellers in the coastal districts of Orissa viz. Kolha, Munda, Santal, Panpano, Shabar, Gokha, Dhoba, Kondhs [9] often depend on honey collection from wild hives as their traditional profession. It provides valuable nutrition in the form of honey, protein-rich pollen and brood for their family. In addition bee products contribute important ingredients of folk and traditional medicine. Melissopalynological analysis and its application may thus improve the economic status of the local people of an area [10], [11]. Approximately 112 families, 370 genera and 632 species of angiosperms represent high floristic diversity in Orissa [12] that can be exploited by the beekeeping industry. As there are only a few small-scale beekeepers in Orissa producing honey for the local market and personal consumption and are hardly sponsored by the Government, only 10 kg of honey on an average is produced per hive-box per year [13].

**Figure 1:**
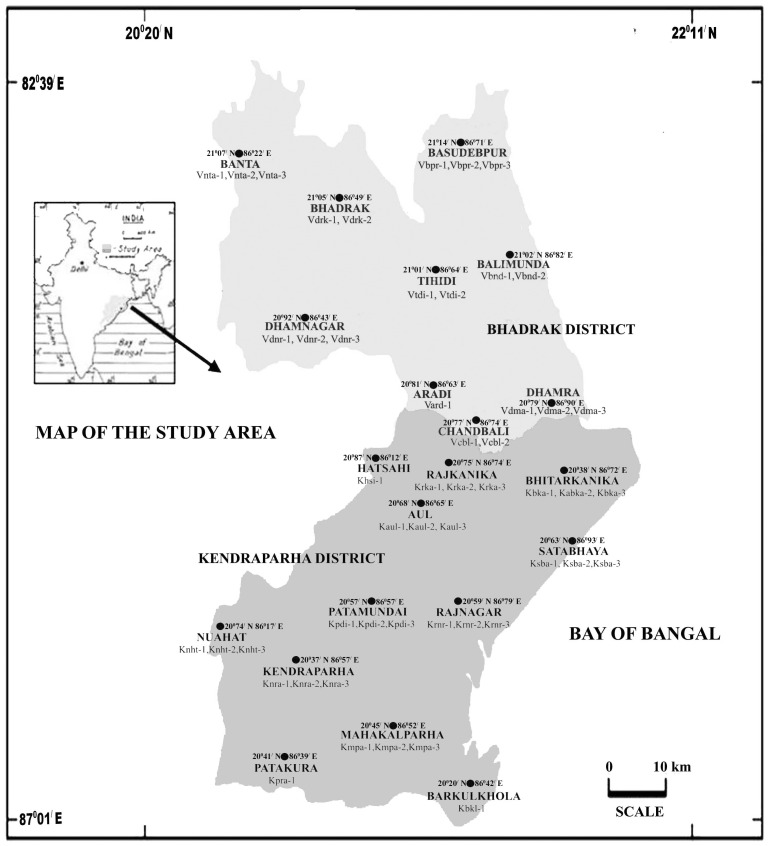
Location Map of study areas in the coastal districts of Orissa, India. Latitudes and longitudes of each study area and honey sample numbers have been mentioned.

Bhadrak and Kendraparha (20^0^20^/^ to 22^0^11^/^ N, 82^0^39^/^ to 87^0^01^/^ E) are two coastal districts of Orissa with dry deciduous and mangrove forests and a vast stretch of coast. These are bounded on the north by the Baleshwar district, Kendujhar, Jajpur and Cuttack districts on the west, Jagatsinghapur district on the south and Bay of Bengal in the east (Fig. 1). The climate of the two coastal districts under study is tropical - moist sub humid to dry sub humid type. It belongs to the mega thermal type with very high temperature during the months of April and May. The rainfall in this area is mostly contributed by the southwest monsoon during the months of June to September [12], [28]. The predominant plants of this area include *Acacia nilotica, Acanthus illicifolius, Aegle marmelos, Aegialitis rotundifolia, Aegiceras corniculatum, Aglaia cucullata, Amaranthus viridis, Antigonon leptopus, Avicennia officinalis, Avicennia marina, Barringtonia acutangula*, *Bauhinia purpurea, Borassus flabellifer, Bruguiera gymnorrhiza, Capparis indica, Ceiba protandra, Ceriops decandra, Clerodendron inerne*, *Cocos nucifera, Crataeva religiosa, Dalbergia spinous, Derris scandens, Diospyros ebenum, Eucalyptus globulus, Eupatorium odoratum, Euphoria longa, Excoecaria agallocha, Fimbristylis falcata, Grewia tiliaefolia*, *Guettarda speciosa*, *Heritiera fomes, H. littoralis, Hibiscus tiliaceus, Hydrophylax maritime, Lagerstroemia parviflora*, *Madhuca indica, Mangifera indica, Merope angulata, Mikania scandens, Mimosa pudica, Myriostachya wightiana, Ocimum sanctum, Pandanas tectorius, Phoenix paludosa*, *Phoenix sylvestris, Phyllanthus rotundifolius, Pongamia pinnata, Prosopis juliflora, Psidium guajava*, *Rhizophora apiculata, R. mucronata, R. stylosa, Saraca asoka, Salmalia malabarica, Scleichera oleosa, Sonneratia apetala, Spinifex littoreus, Syzygium cumini, Spondias dulcis, Tamarix indica, Terminalia arjuna, Thespesia acutifolia, Tridax procumbens, Xylocarpus mekongensis* and *Zizyphus jujuba* [12], [28]. Honey pollen analytical data of this region is lacking and little is known from the whole east coastal India [10], [11], [14], [15]. In order to rectify this insufficiency of melissopalynological database the present study of qualitative and quantitative pollen analysis of natural honey samples from these two districts of Orissa has been undertaken.

## Materials and Methods

### Ethics statement

No specific permits were required for the described field studies. The sampling sites are not protected in any way and the field studies did not involve endangered or protected species.

### Materials

Fifty-one natural honey samples (out of which thirty five were squeezed and sixteen were from apiary) were collected from twenty-one different areas of Bhadrak and Kendraparha districts during different seasons in 2010–2011 (Fig. 1). The honey samples were obtained directly from the honey-bearing portion of the combs of *Apis cerana indica* (31 honey samples), *A. dorsata*, (12 samples) *and A. florea* (8 samples) as far as possible. The honey samples were mostly analyzed immediately after collection. The pH of each sample was measured with the help of a digital pH meter (GLOBAL DPH 500, India). The color of the honey samples was determined by a simple color grading method [16]. An inventory of the honey samples is presented in Table 1.

**Table 1:** Melissopalynological data of natural honey samples from two east-coastal districts of India.

Honey samples, extraction source area and month of collection	pH & Color of the honey samples	Floristic nature, Bee species and the season to which honey samples belong	APC (/10 g) & Group of honey	HDE/P and toxic pollen grains, if any	Bee-plant Species/Melissopalynotaxa (and their Families) recovered	No. of pollen grains & frequency of their occurrence (in %)
1.Vnta-1 (Apiary, Banta, July)	5.9, Yellowish brown	Multifloral, *A. cerana indica*, Summer	18500, I	14/227 = 0.06	*Syzygium cumini* (Myrtaceae)	96 (42.29)
					*Barringtonia acutangula* (Lecythidaceae)	49 (21.59)
					*Saraca asoka* (Fabaceae)	21 (9.25)
					*Ocimum sanctum* (Lamiaceae)	03 (1.32)
					*Alternanthera sessilis* (Amarananthaceae)	12 (5.29)
					*Mimosa pudica* (Fabaceae)	17 (7.49)
					*Borassus flabellifer* (Arecaceae)	29 (12.78)
					Total Number of Pollen grains counted	227
2.Vnta-2 (Squeezed, Banta, April)	6.2, Yellowish brown	Multifloral, *A. dorsata*, Spring	95500, II	29/662 = 0.04	*Tecoma stans* (Bignoniaceae)	114 (17.22)
					*Sesamum indicum* (Pedaliaceae)	27 (4.08)
					*Eucalyptus globulus* (Myrtaceae)	106 (16.01)
					*Syzygium cumini* (Myrtaceae)	235 (35.50)
					*Punica granatum* (Punicaceae)	19 (2.87)
					*Borassus flabellifer* (Arecaceae)	21(3.17)
					*Barringtonia acutangula* (Lecythidaceae)	08 (1.21)
					*Jasminum oleoides* (Oleaceae)	29 (4.38)
					*Prosopis juliflora* (Fabaceae)	16 (2.42)
					*Heliotropium indicum* (Boraginaceae)	25 (3.78)
					*Aegle marmelos* (Rutaceae)	62 (9.37)
					Total Number of Pollen grains counted	662
3.Vnta-3 (Apiary, Banta, January)	6.3, Amber	Unifloral, *A. florea*, Winter	71200, II	36/696 = 0.05	*Tagetes patula* (Asteraceae)	36 (5.17)
					*Mimosa pudica* (Fabaceae)	473 (67.96)
					*Eupatorium odoratum* (Asteraceae)	102 (14.66)
					*Crataeva religiosa* (Capparidaceae)	72 (10.34)
					*Zizyphus jujuba* (Rhamnaceae)	13 (1.87)
					Total Number of Pollen grains counted	696
4.Vdma-2 (Squeezed, Dhamra, May)	6.1, Light Amber	Unifloral, *A. cerana indica*, Spring	63900, II	21/598 = 0.03	*Cocos nucifera* (Arecaceae)	23 (3.85)
					*Borassus flabellifer* (Arecaceae)	26 (4.35)
					*Anacardium occidentale* (Anacardiaceae)	77 (12.88)
					*Avicennia marina* (Acanthaceae)	429 (71.74)
					*Ceriops decandra* (Rhizophoraceae)	43 (7.19)
					Total Number of Pollen grains counted	598
5.Vbpr-2 (Squeezed, Basudebpur, March)	6.3, Dark brown	Unifloral, *A. cerana indica*, Winter	26800, II	67/188 = 0.35	*Anacardium occidentale* (Anacardiaceae)	58 (30.85)
					*Coriandrum sativum* (Apiaceae)	91 (48.40)
					*Phoenix sylvestris* (Arecaceae)	39 (20.74)
					Total Number of Pollen grains counted	188
6.Vbpr-3 (Squeezed, Basudebpur, May)	5.5, Light yellow	Multifloral, *A. florea*, Spring	25600, II	16/587 = 0.02	*Brassica nigra* (Brassicaceae)	191 (32.54)
					*Scleichera oleosa* (Sapindaceae)	95 (16.18)
					*Cocos nucifera* (Arecaceae)	11 (1.87)
					*Mangifera indica* (Anacardiaceae)	30 (5.11)
					*Eucalyptus globulus* (Myrtaceae)	209 (35.60)
					*Flacourtia indica* (Flacourtiaceae)	41(6.98)
					*Tagetes patula* (Asteraceae)	10 (1.70)
					Total Number of Pollen grains counted	587
7.Vdrk-1 (Squeezed, Bhadrak, April)	5.7, Amber	Multifloral, *A. florea*, Spring	54400, II	48/559 = 0.08	*Phoenix sylvestris* (Arecaceae)	135 (24.15)
					*Cocos nucifera* (Arecaceae)	7 (1.25)
					*Peristrophe bicalyculata* (Acanthaceae)	75 (13.42)
					*Capparis indica* (Capparidaceae)	92 (16.46)
					*Coriandrum sativum* (Apiaceae)	37 (6.62)
					*Salmalia malabarica* (Bombacaceae)	18 (3.22)
					*Mimosa pudica* (Fabaceae)	174 (31.13)
					Poaceae	8 (1.43)
					*Carum copticum* (Apiaceae)	13 (2.33)
					Total Number of Pollen grains counted	559
8.Vcbl-1 (Squeezed Chandbali, July)	6.1, Brown	Unifloral, *A. cerana indica*, Summer	50800, II	41/683 = 0.06	*Citrus grandis* (Rutaceae)	35 (5.20)
					*Papaver somnifera* (Papaveraceae)	28 (4.16)
					*Sonneratia apetala* (Lythraceae)	34 (5.05)
					*Aegiceras corniculatum* (Myrsinaceae)	575 (85.56)
					Total Number of Pollen grains counted	672
9.Vtdi-1 (Apiary, Tihidi, February)	6.0, Reddish brown	Unifloral, *A. cerana indica*, Winter	380000, III	7/512 = 0.01	*Mimosa pudica* (Fabaceae)	468 (91.41)
					*Zizyphus jujuba* (Rhamnaceae)	44 (8.59)
					Total Number of Pollen grains counted	512
10.Vtdi-2 (Squeezed, Tihidi, April)	5.7, Yellow	Multifloral, *A. dorsata*, Spring	23000, II	8/519 = 0.01	*Phoenix sylvestris* (Arecaceae)	82 (15.80)
					*Mimosa pudica* (Fabaceae)	22 (4.24)
					*Cocos nucifera* (Arecaceae)	11 (2.12)
					*Psidium guajava* (Myrtaceae)	123 (23.70)
					*Coriandrum sativum* (Apiaceae)	18 (3.47)
					*Eupatorium odoratum* (Asteraceae)	12 (2.31)
					*Spondias dulcis* (Anacardiaceae)	87 (16.76)
					*Euphoria longa* (Sapindaceae)	26 (5.01)
					*Prosopis juliflora* (Fabaceae)	18 (3.47)
					*Jasminum oleoides* (Oleaceae)	30 (5.78)
					*Murraya paniculata* (Rutaceae)	39 (7.51)
					*Zizyphus jujuba* (Rhamnaceae)	51 (9.83)
					Total Number of Pollen grains counted	519
11.Vbnd-1 (Apiary, Balimunda, May)	6.2, Brown	Multifloral, *A. cerana indica*, Spring	7400, I	6/69 = 0.08	*Brassica nigra* (Brassicaceae)	8 (11.59)
					*Portulaca oleracea* (Portulaccaceae)	13 (18.84)
					*Madhuca indica* (Sapotaceae)	29 (42.03)
					*Phoenix sylvestris* (Arecaceae)	5 (7.25)
					*Jasminum oleoides* (Oleaceae)	12 (17.39)
					Poaceae	2 (2.90)
					Total Number of Pollen grains counted	69
12.Vbnd-2 (Squeezed, Balimunda, February)	5.9, Amber	Unifloral, *A. cerana indica*, Winter	8100, I	2/61 = 0.03	*Mimosa pudica* (Fabaceae)	11 (18.03)
					*Madhuca indica* (Sapotaceae)	16 (26.23)
					*Tecoma stans* (Bignoniaceae)	3 (4.92)
					*Zizyphus jujuba* (Rhamnaceae)	31 (50.82)
					Total Number of Pollen grains counted	61
13.Vdnr-1 (Squeezed, Dhamnagar, May)	5.5, Light yellow	Unifloral, *A. dorsata*, Spring	75900, II	14/579 = 0.02	*Syzygium cumini* (Myrtaceae)	487 (84.11)
					*Cocos nucifera* (Arecaceae)	9 (1.55)
					*Coriandrum sativum* (Apiaceae)	13 (2.25)
					*Jasminum oleoides* (Oleaceae)	15 (2.59)
					*Callistemon citrinus* ((Myrtaceae)	41 (7.08)
					*Tridax procumbens* (Asteraceae)	14 (2.42)
					Total Number of Pollen grains counted	579
14.Vdnr-2 (Apiary, Dhamnagar, July)	5.9, Yellowish amber	Unifloral, *A. cerana indica*, Summer	16200, I	12/440 = 0.02	*Syzygium cumini* (Myrtaceae)	412 (93.64)
					*Bauhinia purpurea* (Fabaceae)	14 (3.18)
					*Barringtonia acutangula* (Lecythidaceae)	14 (3.18)
					Total Number of Pollen grains counted	440
15.Vdnr-3 (Squeezed, Dhamnagar, June)	5.5, Yellowish brown	Multifloral, *A. dorsata*, Winter	56800, II	14/648 = 0.02	*Eucalyptus gobulus* (Myrtaceae)	104 (16.05)
					*Cocos nucifera* (Arecaceae)	34 (5.25)
					*Borassus flabellifer* (Arecaceae)	34 (5.25)
					*Coriandrum sativum* (Apiaceae)	128 (19.75)
					*Rosa chinensis* (Rosaceae)	6 (0.93)
					*Salmalia malabarica* (Bombacaceae)	49 (7.56)
					*Carum copticum* (Apiaceae)	55 (8.49)
					*Lagerstroemia parviflora* (Lythraceae)	142 (21.91)
					*Spondias dulcis* (Anacardiaceae)	20 (3.09)
					*Sterculia foetida* (Sterculiaceae)	76 (11.73)
					Total Number of Pollen grains counted	648
16.Vard-1 (Apiary, Aradi, May)	5.3, Light brown	Unifloral, *A. cerana indica*, Spring	35500, II	18/586 = 0.03	*Syzygium cumini* (Myrtaceae)	579 (98.81)
					*Coriandrum sativum* (Apiaceae)	7 (1.19)
					Total Number of Pollen grains counted	586
17.Vdrk-2 (Squeezed, Bhadrak, December)	5.6, Yellow	Unifloral, *A. dorsata*, Winter	81000, II	10/674 = 0.01	*Cleome viscosa* (Cappaidaceae)	167 (24.77)
					*Grewia tiliaefolia* (Tiliaceae)	35 (5.19)
					*Anacardium occidentale* (Anacardiaceae)	15 (2.22)
					*Carum copticum* (Apiaceae)	10 (1.48)
					*Salmalia malabarica* (Malvaceae)	04 (0.59)
					*Alstonia scholaris* (Apocynaceae)	396 (58.75)
					*Schleichera oleosa* (Sapindaceae)	47 (6.97)
					Total Number of Pollen grains counted	674
18.Vcbl-2 (Squeezed, Chandbali, April)	4.9, Amber	Unifloral, *A. cerana indica*, Summer	166000, III	5/608 = 0.01	*Anacardium occidentale* (Anacardiaceae)	32 (5.36)
					*Aegiceras corniculatum* (Myrsinaceae)	457 (76.67)
					*Sonneratia apetala* (Lythraceae)	59 (9.89)
					*Ceiba protandra* (Bombacaceae)	27 (4.53)
					*Pongamia pinnata* (Fabaceae)	21 (3.52)
					Total Number of Pollen grains counted	596
19.Vdma-1 (Squeezed, Dhamra, February)	5.2, Light brown	Unifloral, *A. cerana indica*, Winter	526500I, V	4/665 = 0.01	*Eucalyptus gobulus* (Myrtaceae)	05 (0.75)
					*Cocos nucifera* (Arecaceae)	03 (0.45)
					*Bruguiera gymnorrhiza* (Rhizophoraceae)	649 (97.59)
					*Sonneratia apetala* (Lythraceae)	8 (1.20)
					Total Number of Pollen grains counted	665
20.Vbpr-1 (Apiary, Basudebpur, July)	5.7, Brown	Unifloral, *A. cerana indica*, Spring	64900, II	48/530 = 0.09	*Borassus flabellifer* (Arecaceae)	6 (1.13)
					*Pongamia pinnata* (Fabaceae)	429 (80.94)
					*Coriandrum sativum* (Apiaceae)	21 (3.96)
					*Phoenix sylvestris* (Arecaceae)	7 (1.32)
					*Anacardium occidentale* (Anacardiaceae)	24 (4.52)
					*Mikania scandens* (Asteraceae)	13 (2.45)
					*Phlogacanthus thyrsiflorus* (Acanthaceae)	15 (2.83)
					Total Number of Pollen grains counted	530
21.Vdma-3 (Squeezed, Dhamra, July)	5.6, Light brown	Unifloral, *A. cerana indica*, Spring	93000, II	0/573 = 0.00	Fabaceae	36 (6.28)
					*Acanthus ilicifolius* (Acanthaceae)	537 (93.71)
					Total Number of Pollen grains counted	573
22.Khsi–1 (Squeezed, Hatsahi, June)	5.5, Light amber	Unifloral, *A. florea*, Spring	150100, III	6/568 = 0.01	*Eucalyptus globulus* (Myrtaceae)	486 (85.56)
					*Antigonon leptopus* (Polygonaceae)	36 (6.33)
					*Amaranthus viridis* (Amaranthaceae)	46 (8.09)
					Total Number of Pollen grains counted	568
23.Krka-1 (Apiary, Rajkanika, July)	4.8, Yellowish brown	Unifloral, *A. cerana indica*, Spring	98000, II	7/536 = 0.01	*Syzygium cumini* (Myrtaceae)	289 (53.91)
					*Grewia tiliaefolia* (Tiliaceae)	231 (43.09)
					*Ocimum sanctum* (Lamiaceae)	16 (2.98)
					Total Number of Pollen grains counted	536
24.Krka-2 (Squeezed, Rajkanika, May)	5.0, Yellowish amber	Unifloral, *A. dorsata*, Spring	89500, II	4/482 = 0.01	*Syzygium cumini* (Myrtaceae)	367 (76.14)
					*Ocimum sanctum* (Lamiaceae)	83 (17.21)
					*Barringtonia acutangula* (Lecythidaceae)	32 (6.63)
					Total Number of Pollen grains counted	482
25.Krka-3 (Squeezed, Rajkanika, July)	5.5, Brown	Unifloral, *A. cerana indica*, Summer	95000, II	4/545 = 0.01	*Sonneratia apetala* (Lythraceae)	138 (25.32)
					*Aegiceras corniculatum* (Myrsinaceae)	366 (67.15)
					*Ceiba protandra* (Bombacaceae)	41 (7.52)
					Total Number of Pollen grains counted	545
26.Kaul-1 (Apiary, Aul, July)	5.4, Light brown	Unifloral, *A. cerana indica*, Summer	17600, I	9/158 = 0.05	*Eucalyptus globulus* (Myrtaceae)	35 (22.15)
					*Borassus flabellifer* (Arecaceae)	123 (77.84)
					Total Number of Pollen grains counted	158
27.Kaul-2 (Squeezed, Aul, March)	4.7, Light yellow	Unifloral, *A. florea*, Winter	60400, II	39/379 = 0.10	*Brassica nigra* (Brassicaceae)	243 (64.11)
					*Mimosa pudica* (Fabaceae)	42 (11.08)
					*Eupatorium odoratum* (Asteraceae)	94 (24.80)
					Total Number of Pollen grains counted	379
28.Kaul-3 (Apiary, Aul, July)	5.5, Amber	Unifloral, *A. cerana indica*, Spring	196000, III	14/576 = 0.02	*Mimosa pudica* (Fabaceae)	465 (80.72)
					*Cocos nucifera* (Arecaceae)	74 (12.84)
					*Phyllanthus emblica* (Euphorbiaceae)	37 (6.42)
					Total Number of Pollen grains counted	576
29.Krnr-1 (Squeezed, Rajnagar, February)	5.3, Light brown	Multifloral, *A.dorsata*, Spring	210000, III	6/545 = 0.01	*Aegialitis rotundifolia* (Plumbaginaceae)	19 (3.48)
					*Bruguiera gymnorrhiza* (Rhizophoraceae)	203 (37.24)
					*Phoenix paludosa* (Arecaceae)	238 (43.66)
					*Casuarina equisetifolia* (Casuarinaceae)	85 (15.59)
					Total Number of Pollen grains counted	545
30.Krnr-2 (Squeezed, Rajnagar, April)	4.6, Yellowish brown	Unifloral, *A. dorsata*, Spring	240000, III	11/595 = 0.01	*Bruguiera gymnorrhiza* (Rhizophoraceae)	522 (87.73)
					*Solanum melongena* (Solanaceae)	56 (9.41)
					Unidentified	17 (2.85)
					Total Number of Pollen grains counted	595
31.Krnr-3 (Squeezed, Rajnagar, June)	5.4, Yellowish amber	Unifloral, *A. cerana indica*, Summer	109000, III	9/472 = 0.01	*Syzygium cumini* (Myrtaceae)	90 (19.06)
					*Aegiceras corniculatum* (Myrsinaceae)	142 (30.08)
					*Phoenix paludosa* (Arecaceae)	240 (50.84)
					Total Number of Pollen grains counted	472
32.Kbka-1 (Squeezed, Bhitrkanika, July)	5.1, Amber	Unifloral, *A. cerana indica*, Summer	515000, IV	0/565 = 0.00	*Aegiceras corniculatum* (Myrsinaceae)	516 (91.32)
					Unidentified	49 (8.67)
					Total Number of Pollen grains counted	565
33.Kbka-2 (Squeezed, Bhitrkanika, February	5.5, Yellowish brown	Unifloral, *A.cerana indica*, Winter	296000, III	18/395 = 0.04	*Bruguiera gymnorrhiza* (Rhizophoraceae)	199 (50.37)
					*Derris scandens* (Fabaceae)	87 (22.02)
					*Phoenix paludosa* (Arecaceae)	66 (16.70)
					*Amaranthus viridis* (Amaranthaceae)	43 (10.88)
					Total Number of Pollen grains counted	395
34.Kbka-3 (Squeezed, Bhitrkanika, June	4.9, Amber	Multifloral *A. cerana indica* Spring	96500, II	0/461 = 0.00	*Bruguiera gymnorrhiza* (Rhizophoraceae)	193 (41.86)
					*Derris scandens* (Fabaceae)	100 (21.69)
					*Aegialitis rotundifolia* (Plumbaginaceae)	168 (36.44)
					Total Number of Pollen grains counted	461
35.Ksba-1 (Squeezed, Satabhaya, April	5.6, Yellowish brown	Unifloral, *A. dorsata*, Spring	195000, III	3/573 = 0.005	*Derris scandens* (Fabaceae)	63 (10.99)
					*Bruguiera gymnorrhiza* (Rhizophoraceae)	212 (36.99)
					*Sonneratia apetala* (Lythraceae)	298 (52.00)
					Total Number of pollen grains counted	573
36.Ksba-2 (Squeezed, Satabhaya, February)	4.7, Light brown	Unifloral, *A. cerana indica*, Spring	190000, III	0/551 = 0.00	*Bruguiera gymnorrhiza* (Rhizophoraceae)	202 (36.66)
					*Phoenix paludosa* (Arecaceae)	302 (54.80)
					*Amaranthus viridis* (Amaranthaceae)	47(8.52)
					Total Number of Pollen grains counted	551
37.Ksba-3 (Squeezed, Satabhaya, June)	5.6, Light amber	Unifloral, *A. cerana indica*, Summer	78000, II	2/402 = 0.004	*Aegiceras corniculatum* (Myrsiniaceae)	129 (32.08)
					*Sonneratia apetala* (Lythraceae)	246 (61.19)
					*Amaranthus viridis* (Amaranthaceae)	27 (6.71)
					Total Number of Pollen grains counted	402
38.Kpdi-1 (Squeezed, Patamundai, June)	4.8, Yellowish brown	Unifloral, *A. dorsata*, Summer	13000, I	05/433 = 0.02	*Eucalyptus globulus* (Myrtaceae)	83 (19.16)
					*Cocos nucifera* (Arecaceae)	34 (7.85)
					*Borassus flabellifer* (Arecaceae)	316 (72.97)
					Total Number of Pollen grains counted	433
39.Kpdi-2 (Squeezed, Patamundai, April)	6.4, Light yellow	Multifloral, *A. dorsata,* Spring	17500, I	09/352 = 0.02	*Eucalyptus globulus* (Myrtaceae)	26 (7.38)
					*Coriandrum sativum* (Apiaceae)	102 (28.97)
					*Madhuca indica* (Sapotaceae)	46 (13.06)
					*Rosa chinensis* (Rosaceae)	38 (10.79)
					*Phoenix sylvestris* (Arecaceae)	31 (8.80)
					*Moringa oleifera* (Moringaceae)	43 (12.21)
					*Butea monosperma* (Fabaceae)	66 (18.75)
					Total Number of Pollen grains counted	352
40.Kpdi-3 (Squeezed, Patamundai, March)	5.7, Yellowish brown	Unifloral, *A. florea,* Spring	74000, II	03/531 = 0.01	*Eucalyptus globulus* (Myrtaceae)	268 (50.47)
					*Mimosa pudica* (Fabaceae)	79 (14.87)
					*Borassus flabellifer* (Arecaceae)	33 (6.21)
					*Antigonon leptopus* (Polygonaceae)	87 (16.38)
					*Amaranthus viridis* (Amaranthaceae)	40 (7.53)
					*Phoenix sylvestris* (Arecaceae)	24 (4.51)
					Total Number of Pollen grains counted	531
41.Knht-1 (Apiary, Nuahat, June)	6.1, Light brown	Multifloral, *A. cerana indica,* Summer	81500, II	6/567 = 0.01	*Pongamia pinnata* (Fabaceae)	194 (34.21)
					*Grewia tiliaefolia* (Tiliaceae)	108 (19.04)
					*Amaranthus viridis* (Amaranthaceae)	28 (4.93)
					*Madhuca indica* (Sapotaceae)	55 (9.70)
					*Borassus flabellifer* (Arecaceae)	36 (6.34)
					*Punica granum* (Punicaceae)	64 (11.28)
					*Capparis indica* (Capparidaceae)	82 (14.46)
					Total Number of Pollen grains counted	567
42.Knht-2 (Apiary, Nuahat, March)	6.0, Light yellow	Unifloral, *A. cerana indica,* Winter	96000, II	2/511 = 0.003	*Eucalyptus globulus* (Myrtaceae)	357 (72.26)
					*Capparis indica* (Capparidaceae)	64 (12.95)
					Poaceae	73 (14.77)
					Total Number of Pollen grains counted	494
43.Knht-3 (Apiary, Nuahat, May)	5.6, Amber	Multifloral, *A. cerana indica,* Summer	155000, III	1/526 = 0.002	*Eucalyptus globulus* (Myrtaceae)	219 (41.63)
					*Grewia tiliaefolia* (Tiliaceae)	218 (41.44)
					*Amaranthus viridis* (Amaranthaceae)	21 (3.99)
					*Cleome viscosa* (Capparidaceae)	68 (12.92)
					Total Number of Pollen grains counted	526
44.Knra-1 (Squeezed, Kendrapara, February)	4.7, Light yellow	Multifloral, *A. florea,* Winter	14500, I	17/382 = 0.04	*Mimosa pudica* (Fabaceae)	102 (26.70)
					*Brassica nigra* (Brassicaceae)	105 (27.48)
					*Phoenix sylvestris* (Arecaceae)	97 (25.39)
					*Mikania scandens* (Asteraceae)	36 (9.42)
					*Capsicum frutescence* (Solanaceae)	42 (10.99)
					Total Number of Pollen grains counted	382
45.Knra-2 (Squeezed, Kendrapara, April)	5.5, Amber	Unifloral, *A. cerana indica,* Spring	533000, IV	0/547 = 0.00	*Phoenix paludosa* (Arecaceae)	452 (82.63)
					*Amaranthus viridis* (Amaranthaceae)	27 (4.93)
					*Lycopersicon esculentum* (Solanaceae)	68 (12.43)
					Total Number of Pollen grains counted	547
46.Knra-3 (Apiary, Kendrapara, July)	4.9, Light amber	Unifloral, *A. cerana indica,* Summer	503000, IV	3/526 = 0.01	*Terminalia arjuna* (Combretaceae)	319 (60.64)
					*Capsicum frutescence* (Solanaceae)	32 (6.08)
					*Acacia nilotica* (Fabaceae)	14 (2.66)
					*Tamarindus indica* (Fabaceae)	27 (5.13)
					*Mimusops elengi* (Sapotaceae)	33 (6.27)
					*Lagerstroemia parviflora* (Lythraceae)	101 (19.20)
					Total Number of Pollen grains counted	526
47.Kmpa-1 (Apiary, Mahakalpara, June)	5.4, Amber	Unifloral, *A. cerana indica,* Summer	45000, II	4/515 = 0.01	*Eucalyptus globulus* (Myrtaceae)	423 (82.13)
					*Amaranthus viridis* (Amaranthaceae)	19 (3.68)
					*Phoenix sylvestris* (Arecaceae)	73 (14.17)
					Total Number of Pollen grains counted	515
48.Kmpa-2 (Squeezed, Mahaklpara, May)	5.2, Light brown	Unifloral, *A. florea*, Spring	105000, II	7/424 = 0.01	*Borassus flabellifer* (Arecaceae)	92 (21.69)
					*Antigonon leptopus* (Polygonaceae)	103 (24.29)
					*Coriandrum sativum* (Apiaceae)	24 (5.66)
					*Prosopis juliflora* (Fabaceae)	205 (48.34)
					Total Number of Pollen grains counted	424
49.Kmpa-3 (Squeezed, Mahaklpara, February)	6.0, Light yellow	Multifloral, *A. florea,* Winter	59500, II	21/426 = 0.04	*Eucalyptus globulus* (Myrtaceae)	19 (4.46)
					*Coriandrum sativum* (Apiaceae)	183 (42.95)
					*Brassica nigra* (Brassicaceae)	103 (24.17)
					*Cleome viscosa* (Capparidaceae)	27 (6.33)
					*Zizyphus jujuba* (Rhamnaceae)	74 (17.37)
					*Ailanthus excelsa* (Simaroubaceae)	20 (4.69)
					Total Number of Pollen grains counted	426
50.Kpra-I (Squeezed, Patakura, May)	5.6, Light yellow	Unifloral, *A. dorsata,* Summer	64000, II	22/574 = 0.03	*Cocos nucifera* (Arecaceae)	574 (100)
					Total Number of Pollen grains counted	574
51.Kbkl-1 (Apiary, Barkulkhla, April)	5.5, Light brown	Unifloral, *A. cerana indica,* Spring	16000, I	06/509 = 0.01	*Cocos nucifera* (Arecaceae)	461 (90.56)
					*Madhuca indica* (Sapotaceae)	48 (9.43)
					Total Number of Pollen grains counted	509

### Methods

For qualitative melissopalynogical analysis, honey samples were processed by acetolysis method adopted by Erdtman [17] with some modifications recommended by the International Commission for Bee Botany [1], [2]. Ten grams of each honey sample were transferred to a pointed glass centrifuge tube (50 ml capacity), stirred with a 1 ml pipette and dissolved in 20 ml of distilled water (20–40°C) and centrifuged for 10 min at 2000 *g*. The supernatant was decanted off. The process was repeated twice. The supernatant liquid was decanted off and all but the last drop is removed by placing the tube upside down at a 45° angle to allow the remaining excess liquid to be taken up on absorbent paper. The sediment obtained was treated with acetolysis mixture (acetic anhydride: conc. sulphuric acid  = 9∶1 v/v). The centrifuge tube with acetolysed pollen sediment was placed in a 70°C water bath for 10 min. After incubation the tube was centrifuged for 5 min and decanted carefully into a dry beaker. The tube was filled with distilled water and a drop of a strong detergent added and shaken vigorously and centrifuged again for 5 min. The supernatant liquid was drawn off. The entire sediment was distributed more or less equally on three slides and spread out over an area of about 20×20 mm. A drop of glycerin jelly was applied to the sediment in each slide. Another drop was poured on a cover glass, which was placed on the sediment-glycerin jelly mixture. To analyze the pollen content three pollen slides were prepared in this way from each honey sample studied and photographed under a compound microscope (Zeiss, Axioskop 2). Dried acetolysed pollen sediments were also photographed using a Scanning Electron Microscope (Carl Zeiss, Evo 40). The pollen grains were identified with the help of reference slides prepared from a collection of the local flora (Repository of spore-pollen slides of modern taxa, CUH, Palaeobotany and Palynology section, Center of Advanced Studies, Department of Botany, and University of Calcutta) and published literature [18], [19], [20], [14], [15], [21], [22]. The majority of the pollen types could be identified at the species level and those types that could not be identified were mentioned as unidentified.

For quantification of pollen grain types 500 pollen grains were randomly counted from each sample. Percentage frequency of the pollen taxa in all the samples was calculated. Unacetolysed honey samples were examined for computing the (HDE/P) ratio of Honey Dew Elements (HDE) and total number of pollen grains (P) of bee-plant taxa in each sample. Recommendations of the International Commission for Bee Botany [1] were followed for determination of the frequency classes. Pollen frequency classes are determined as predominant pollen (represented by >45% of the pollen grains counted), secondary pollen (16–45%), important minor pollen (3–15%) and minor pollen (less than 3%). A honey sample with one predominant pollen type was regarded as unifloral (and multifloral otherwise).

The absolute pollen count (APC) of the honey samples (i.e. the number of pollen grains per 10 g of honey) was calculated using a haemocytometer [23]. The samples were categorized under various groups in conformity with the universally followed grading parameters [1] viz., Group I (<20,000 pollen grains), Group II (20,000–100,000), Group III (100,000–500,000), Group IV (500,000–1,000,000) and Group V (>1,000,000).

A nectar calendar of the study area was constructed from the palynotaxa frequency data by considering all predominant pollen types in the unifloral honeys as chief nectar sources and all pollen types represented by at least 10% in the honey samples as alternative nectar sources. The seasons when a large amount of nectar from flowers is available are Honey Flow Period while the seasons when nectar source is limited and/or not available to the honey bees (for example due to heavy rain during the rainy season bee forage is affected seriously) are Honey Dearth Period.

We have attempted a multivariate analysis through Principal Component Analysis (PCA) of the melissopalynological data to evaluate the classification of these natural honey samples according to their botanical origin. Quality control methods, in conjunction with multivariate statistical analysis, have proved to be potent for classifying honey from different geographic regions [24], [25]. The matrix comprised the absolute values of all the parameters measured for the taxa recorded in each sample. PAST 2.17 version 3.0.9 [26] is used for principal component analysis (PCA) from the pollen data that are explored to derive the first principal components which are further analyzed by Microcal Origin version 6.0 [27] to examine the grouping of the samples in order to understand the relative distribution of unifloral honey samples according to their botanical origin.

## Results

**Figure 2:**
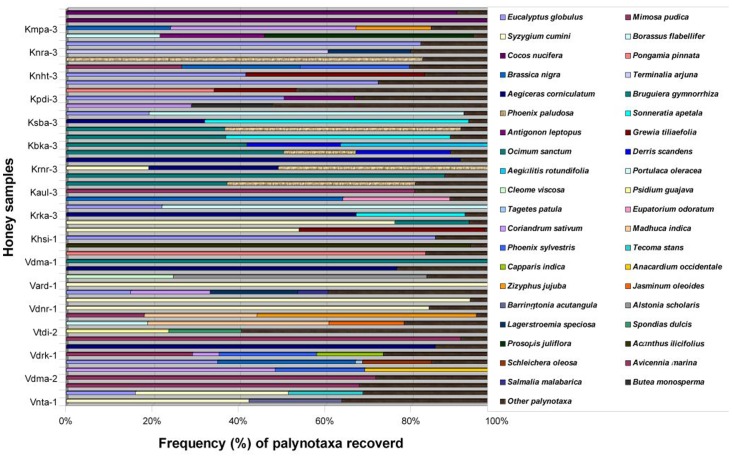
Frequency (%) of palynotaxa recovered.

**Figure 3:**
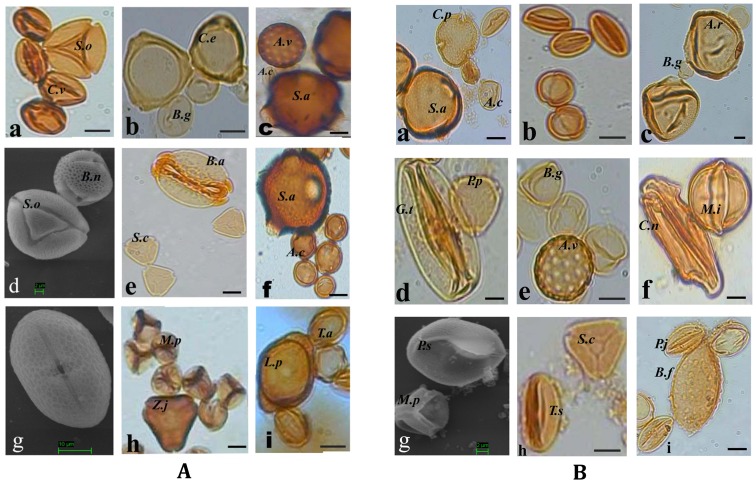
A) Photomicrographs and scanning electron micrographs of some of the significant and characteristic pollen assemblage recovered from the natural honey samples of coastal district of Bhadrak and Kendraparha, India. a. *Schleichera oleosa* (*S.o*) and *Cleome viscosa* (*C.v*) found in sample Vdrk-2; b. *Casuarina equisetifolia* (*C.e*) and *Bruguiera gymnorrhiza* (*B.g*) found in sample Krnr-1; c. *Sonneratia apetala* (*S.a*), *Aegiceras corniculatum* (*A.c*) and *Amaranthus viridis* (*A.v*) found in sample Ksba-3; d. *Brassica nigra* (*B.n*) and *Scleichera oleosa* (*S.o*) in sample Vbpr-3; e. *Syzygium cumini* (*S.c*) and *Barringtonia acutangula* (*B.a*) in sample Krka-2; f. *Aegiceras corniculatum* (*A.c*) and *Sonneratia apetala* (*S.a*) in sample Vcbl-2; g. *Acanthus ilicifolius* (*A.i*) in sample Vdma-3; h. *Mimosa pudica* (*M.p*) and *Zizyphus jujuba* (*Z.j*) in sample Vtdi-1; i. *Terminalia arjuna* (*T.a*) and *Lagerstroemia parviflora* (*L.p*) in sample Knra-3; *B*. a. *Aegiceras corniculatum* (*A.c*), *Ceiba protandra* (*C.p*) and *Sonneratia apetala* (*S.a*) in sample Krka-3; b. *Aegiceras corniculatum* (*A.c*) in sample Kbka-1; c. *Bruguiera gymnorrhiza* (*B.g*) and *Aegialitis rotundifolia* (*A.r*) in sample Kbka-3; d. *Pongamia pinnata* (*P.p*) and *Grewia tiliaefolia* (G.*t*) in sample Knht-1; e. *Bruguiera gymnorrhiza* (*B.g*) and *Amaranthus viridis* (*A.v*) in sample Ksba-2; f. *Cocos nucifera* (*C.n*) and *Madhuca indica* (*M.i*) in sample Kbkl-1; g. *Phoenix sylvestris* (*P.s*) and *Mimosa pudica* (*M.p*) in sample Vdrk-1; h. *Tecoma stans* (*T.s*) and *Syzygium cumini* (*S.c*.) in sample Vnta-2; i. *Prosopis juliflora* (*P.j*) and *Borassus flabellifer* (*B.f*) in sample Kmpa-2. Italicized initials within parenthesis after each palynotaxon above is given (as suggested by the referee) to indicate species level identification of the taxon in the respective figures of *Fig.3*.*A* and *B* Bar  = 10 µm unless otherwise mentioned (original magnification: 400× and 1000×; d and g of Plate 1 and g of Plate 2 are scanning electron micrographs).

Melissopalynological study assesses the status of native flora [4]. On the basis of microscopic analysis of fifty-one honey samples eighty-two angiospermic taxa belonging to forty-four families are demonstrated as bee plants in these coastal districts of India (Table 1). Three species of honeybees viz., *Apis cerana indica*, *A. dorsata* and *A. florea* contribute to these honeys. Thirty-seven samples are proved to be unifloral, twenty-five of which are contributed by *Apis cerana indica*, seven by *A. dorsata* and the remaining five by *A. florea*. Fourteen samples are multifloral, five of which contributed by *A. cerana indica*, five by *A. dorsata* and the remaining four by *A. florea*. The extent to which a given honey is derived from different plant sources can be deduced from the frequencies of the different pollen in it [28]. The predominant nectariferous taxa are *Acanthus ilicifolius, Aegiceras corniculatum, Alstonia scholaris, Avicennia marina, Borassus flabellifer, Brassica nigra, Bruguiera gymnorrhiza, Cocos nucifera, Coriandrum sativum*, *Eucalyptus globulus, Phoenix paludosa*, *Pongamia pinnata, Prosopis juliflora*, *Sonneratia apetala, Syzygium cumini, Terminalia arjuna*, and *Zizyphus jujuba*. Thirty two secondary pollen taxa are recorded among which the significant are *Anacardium occidentale*, *Antigonon leptopus, Barringtonia acutangula*, *Borassus flabellifer, Cleome viscosa*, *Derris scandens, Eupatorium odoratum, Grewia tiliaefolia*, *Lagerstroemia parviflora*, *Madhuca indica, Phoenix sylvestris* and *Psidium guajava* (Table 1). *Mimosa pudica*, *Amaranthus viridis* and Poaceae types do not have nectar, so probably the bees forage these plants as a pollen source or are common probably because they become attached to the honeybees due to the close proximity of the non-nectariferous taxa to nectariferous ones. The absolute Pollen Count (APC) per 10 g of honey samples indicates that most of the samples (approximately 55%) belong to Group II (27 samples) and III (11 samples) 9 samples to Group I while 4 samples belong to Group IV. Absolute count and percentage frequency in all the samples are graphically represented in Fig. 2. HDE/P of the samples ranges from 0.0 to 0.1 with the lowest value (0.0) shown by the samples Vdma-III, Knra-II, Ksba-II, Kbka-I and Kbka-III and highest value (0.1) by Kaul-II (Table 1). The pH of the samples ranges from 4.6 to 6.4 with lowest pH (4.6) shown by the sample Krnr-II and highest pH (6.4) by Kpdi-II. The color range of the samples varies from light yellow (in eight samples) to dark brown (in the sample Vbpr-II). Photomicrographs and scanning electron micrographs of the significant and predominant palynotaxa recovered from the honey samples are recorded and presented in Fig. 3.A and B. Three taxa could not be identified (and are referred to as unidentified).

There are remarkable differences in the overall pollen contents of the honeys of Bhadrak and Kendraparha despite their geographical proximity. Fifty nine percent of the total honey samples are from Kendraparha while 41% of the samples are from Bhadrak. *Aegiceras corniculatum, Bruguiera gymnorrhiza, Mimosa pudica* and *Syzygium cumini* represent the predominant palynotaxa in both districts but *Acanthus ilicifolius*, *Alstonia scholaris, Avicennia marina, Coriandrum sativum*, *Pongamia pinnata* and *Zizyphus jujuba* are found to be predominant palynotaxa in Bhadrak. Whereas *Borassus flabellifer*, *Brassica nigra, Cocos nucifera, Eucalyptus globulus, Phoenix paludosa*, *Prosopis juliflora*, *Sonneratia apetala* and *Terminalia arjuna* are found to be predominant in Kendraparha. All three honeybees *A. cerana indica, A. dorsata and A. florea* prefer arboreal families especially Arecaceae, Myrtaceae and Fabaceae and in the mangrove areas Rhizophoraceae, Myrsinaceae and Lythraceae. *A. dorsata* never forage on *Alternanthera sessilis, Brassica nigra, Cleome gynandra, Cleome viscosa, Mikania scandens* probably due to their feeble and slender peduncles and the small size of these flowers. *A. florea* often forages on herbs near the agricultural fields. HDE/P values (Mean±SD) are lower (0.01±0.02) in Kendraparha samples than those in Bhadrak (0.49±0.07).

**Figure 4:**
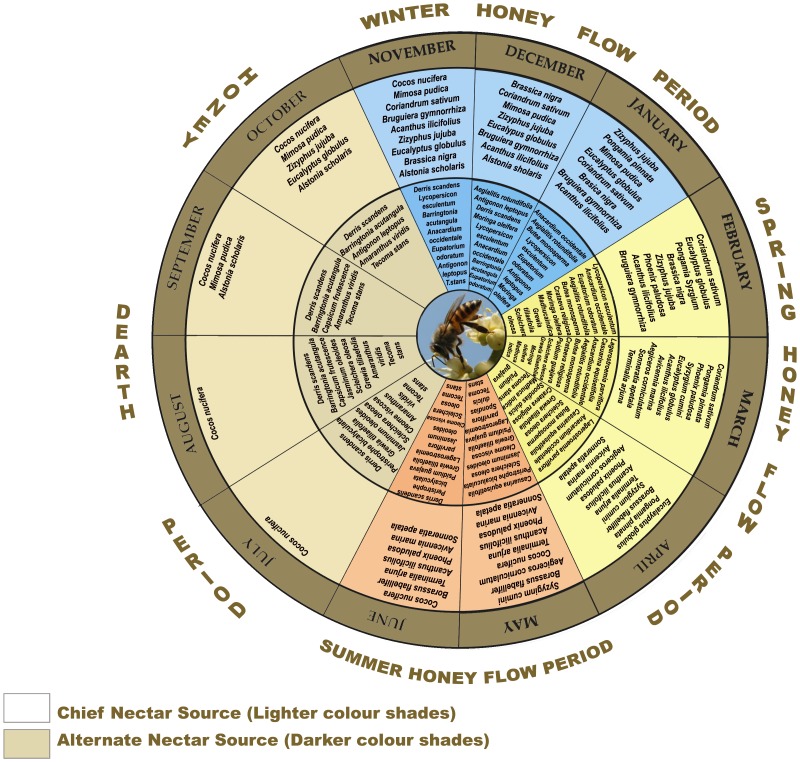
Nectar calendar of Bhadrak and Kendraparha districts, Orissa, India.

**Figure 5:**
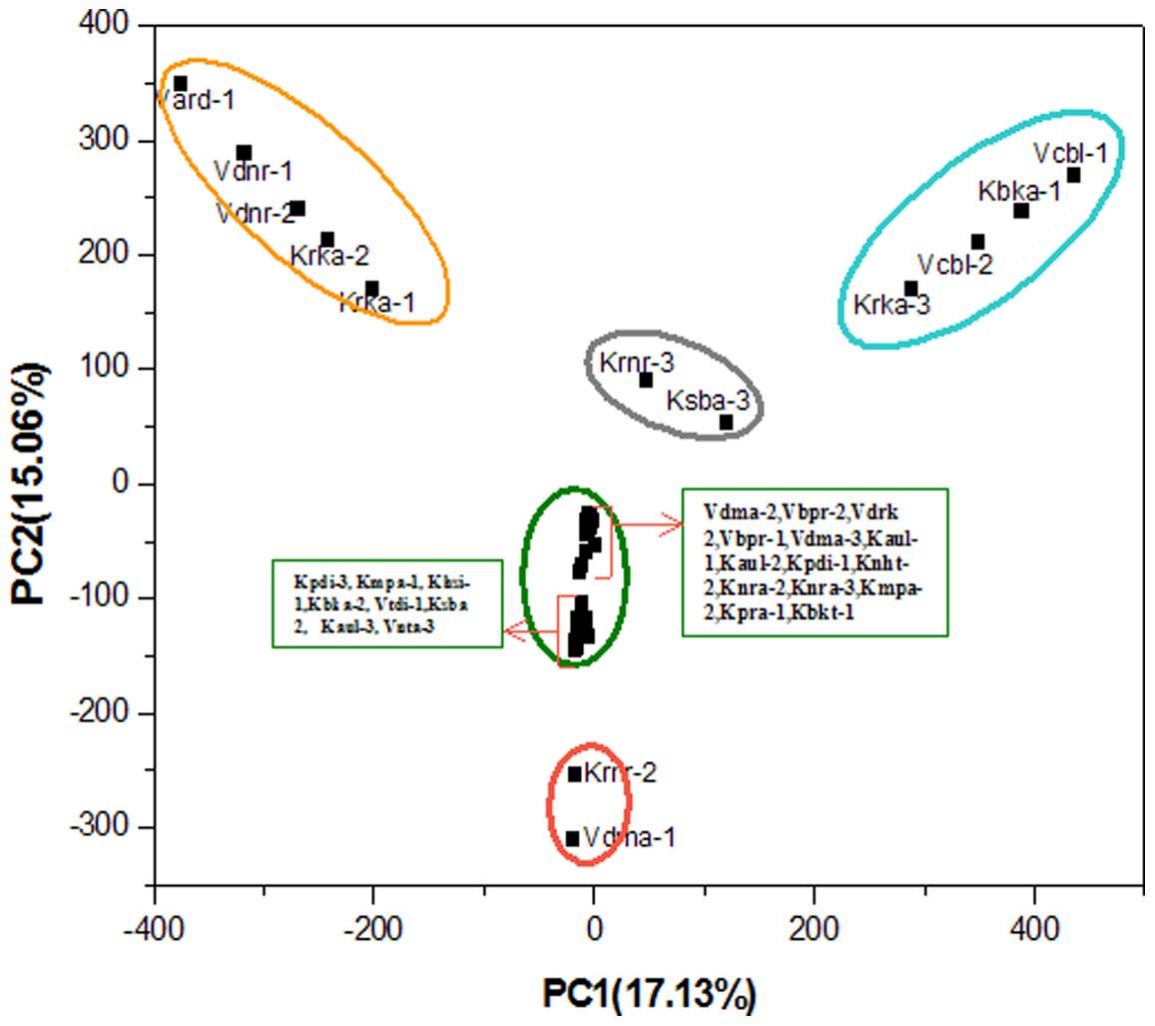
Principal Component Analysis (PCA) of the honey samples using the absolute value variables per sample.

The nectar calendar of the study area shows that the overall honey flow period is from November to June which is divided into a Spring Honey Flow Period (Spring HFP) during February-April, a Summer Honey Flow Period (Summer HFP) from May to June and a Winter Honey Flow Period (Winter HFP) during November- January (Fig. 4). July to October is recognized as the Honey Dearth Period (HDP) because few plants serve as nectar sources possibly due to the heavy monsoon in the area. The score plot of the two principal components (PC1 and PC2) for the classification of honey samples according to their botanical origin is determined (Fig. 5). A distinct separation of honey samples is observed according to their botanical origin. The samples that are grouped in the clusters have pollen grains in common and the samples in this work are clustered into five groups. The left uppermost ellipse is a cluster in which the honey samples with *Syzygium* as predominant pollen type are grouped together. The right uppermost cluster includes the samples with *Aegiceras* as the predominant pollen type, the middle horizontal cluster represents the honey samples with *Aegiceras* as the secondary pollen type and the lowermost cluster includes honey samples with *Bruguiera* as the predominant pollen type while the middle vertical cluster includes all the remaining honey samples without *Syzygium*, *Aegiceras* and *Bruguiera* pollen grains. Principal Component Analysis has confirmed that all of the thirty-seven unifloral honey samples have been classified correctly by the melissopalynological analysis.

## Discussion and Conclusion

Most of the indigenous people are tribes who have collected honey as a livelihood for generations. Due to an absence of organized bee keeping, since 1975 these people collect and sell honey $ 0.40 per kg. Since cultivable land is scanty in this coastal area, 25% of the local tribes depend on fishing, 21% on farming and the remaining 20% of the population are wage-earning laborers in fishing, farming and forest product-related occupations while, nearly 35% people do not have any occupation [29]. Thus there is an urgent need for socioeconomic improvement of this eastern coastal region of India where three-fourths of the population lives under the poverty line [8].

Results of this work reveal that 70% of the honeys are unifloral among which 30% are from the mangrove forests. Principal Component Analysis has confirmed that all the thirty-seven unifloral honey samples have been classified correctly by the melissopalynological analysis. There is a high demand for unifloral honey in the local as well as in the global market [30]. Unifloral nature, very low HDE/P (<0.09 for most of the samples) and absence of toxic palynotaxa guarantee the suitability of these honey samples for human consumption.

Honeybees are effective and potential pollinators in agricultural and natural ecosystems in different seasons; this can enhance the crop productivity improving the overall socio-economic status of the local tribal communities. A monthly nectar calendar of all the honeybee species would educate rural beekeepers to produce unifloral honey in this region. This calendar of the study area and extensive field observations of bee pasturing in the present study reveal that during the Winter HFP, *Alstonia scholaris, Brassica nigra, Bruguiera gymnorrhiza, Coriandrum sativum, Eucalyptus globulus Pongamia pinnata, Syzyzium cumini* and *Zizyphus jujuba* are found to be nectar rewarding to the honeybees. During the Spring HFP the nectariferous taxa are *Acanthus ilicifolius, Avicennia marina, Bruguiera gymnorrhiza, Cocos nucifera, Eucalyptus globulus, Lagerstroemia parviflora, Moringa oleifera, Phoenix paludosa, Pongamia pinnata, Prosopis juliflora*, *Sonneratia apetala* and *Syzygium cumini* while during the Summer HFP the bees collect nectar from *Aegiceras corniculatum, Barringtonia acutangula, Borassus flabellifer, C. nucifera, E. globulus, Phoenix paludosa, Sonneratia apetala, Syzygium cumini, Tamarindus indica*, and *Terminalia arjuna*. Thus the honey flow period in this area is quite extended (November to June) with a relatively short honey dearth period (July to October), which is congenial for sustainable apicultural entrepreneurship.

Among the 82 nectariferous taxa Coriandrum sativum, Eucalyptus globulus, Prosopis juliflora and Terminalia arjuna are medicinally important plants; Borassus flabellifer, Syzygium cumini, and Zizyphus jujuba are fruit yielding plants; Brassica nigra and Cocos nucifera are oil yielding plants while Acanthus ilicifolius, Aegiceras corniculatum, Avicennia marina, Bruguiera gymnorrhiza, Phoenix paludosa, Pongamia pinnata, and Sonneratia apetala are mangrove plants. Arecaceae are represented in 29 samples followed by Myrtaceae and Fabaceae each in 22 and Apiaceae in 12 samples, Anacardiaceae, Rhizophoraceae and Lythraceae each in 8, Myrsinaceae 7 and Asteraceae and Sapotaceae each in 6 samples. Highest number of palynotaxa (12) is recorded in the sample Vtdi-II collected from Tihidi in Bhadrak and lowest number of palynotaxa (1) in Kpra-I collected from Patakura in Kendraparha. The vegetation cover in this region is thus diverse enough to sustain bee colonies. Local vegetation plays a significant role as to which pollens are collected by bees [31] and it is possible to produce several monofloral honeys in a region with great plant diversity [32].

This is the first case study of melissopalynological evaluation of apicultural entrepreneurship in India taking the coastal districts as a model. The findings of melissopalynology point to the potential commercial value of the honey market [4]. Qualitative and quantitative melissopalynological analyses in the present study demonstrate that these coastal regions are rich in bee plants with a potential of producing adequate unifloral honey, have an extended honey flow period and thus can be utilized commercially for a moderate to large-scale apicultural venture. We suggest that the best possible regions for apicultural entrepreneurship may be the areas near cultivated vegetation in Basudevpur, Bhadrak, Tihidi, Dhamnagar in Bhadrak district and Aul, Rajkanika, Patamundai, Kendraparha, Mahakalparha in Kendraparha district; the adjacent dry deciduous forests of Banta and Tihidi in Bhadrak, Nuahat, Patamundai, and Mahakalparha in Kendraparha and the mangrove forests of Dhamra and Chandbali in Bhadrak and Rajkanika, Bhitarkanika, Satavaya and Rajnagar in Kendraparha are also suitable for this venture. The competent authority, on the basis of the pollen analytical data presented in this work, should initiate apicultural enterprises in these two eastern coastal districts of India to improve the socio-economy of the local inhabitants of this region.
